# Supplementary material for: Effect of Saccharomyces boulardii Supplementation on Performance and Physiological Traits of Holstein Calves under Heat Stress Conditions
Source: Animals (Basel). 2019 Jul 31;9(8):510. doi: 10.3390/ani9080510 (PMC6719173; doi:10.3390/ani9080510)
Supplement: Supplementary file 1 [file animals-09-00510-s001.pdf]

Table S1. Primers/probe sets used for quantitative real-time PCR.

Fluorescence	Target	Primers	Sequence (5′–3′)	Standard strain	Equation of standard curve (R^2)
SYBR Green-based					
	S. cerevisiae	For<br>Rev	GAGTCGAGTTGTTTGGGAATGC<br>TCTCTTTCCAAAGTTCTTTTCATCTTT	*Sacharomyces cerevisiae*<br>ATCC 7752	Y= -3.397X+39.112<br>(0.999)
	Lactobacillus	For<br>Rev	AGCAGTAGGGAATCTTCCA<br>CACCGCTACACATGGAG	*Lactobacillus acidophilus*<br>ATCC4357	Y= -3.871X+40.112<br>(0.999)
	Prevotella	For<br>Rev	TCAAGTCCTCATGGCCCTTATG<br>CGCCATTGTAGCACGTGTGTA	*Prevotella copri*<br>ATCC 6153	Y= -3.107X+37.324<br>(0.999)
	Enterobacteriaceae	For<br>Rev	TGCCGTAACTTCGGGAGAAGGCA<br>TCAAGGCTCAATGTTCAGTGTC	*Escherichia coli*<br>ATCC 2441	Y= -3.432X+39.94<br>(0.999)
	E. coli	For<br>Rev	CATGCCGCGTGTATGAAGAA<br>CGGGTAACGTCAATGAGCAAA	*Escherichia coli*<br>ATCC 2441	Y= -3.421X+38.81<br>(0.999)
TaqMan probe-based					
	C. perfringens	For<br>Rev<br>Probe	AAAAGAAAGATTTGTAAGGCGCTTAT<br>CCCAAGCGTAGACTTTAGTTGATG<br>FAM–TGCCGCGCTAGCAACTAGCCTATGG–TAMRA	*C. perfringens*<br>*ATCC3624*	Y= -3.523X+38.79<br>(0.999)
	S. enteritidis	For<br>Rev<br>Probe	GGCTTCGGTATCTGGTGGTGTA<br>GGTCATTAATATTGGCCCTGAATA<br>FAM–CCACTGTCCCGTTCGTTGATGGACA–TAMRA	*S.* Enteritidis<br>*KVPH106*	Y= -3.341X+40.13<br>(0.999)
